# A Comparative Study on the Temperature Effect of Solid Birch Wood and Solid Beech Wood under Impact Loading

**DOI:** 10.3390/ma14247616

**Published:** 2021-12-10

**Authors:** Georg Baumann, Reinhard Brandner, Ulrich Müller, Alexander Stadlmann, Florian Feist

**Affiliations:** 1 Faculty of Mechanical Engineering and Economic Sciences, Vehicle Safety Institute, Graz University of Technology, 8010 Graz, Austria; florian.feist@tugraz.at; 2 Faculty of Civil Engineering, Institute of Timber Engineering and Wood Technology, Graz University of Technology, 8010 Graz, Austria; reinhard.brandner@tugraz.at; 3 Department of Material Sciences and Process Engineering (MAP), Institute of Wood Technology and Renewable Materials, BOKU–University of Natural Resources and Life Sciences, 3430 Tulln an der Donau, Austria; ulrich.mueller@boku.ac.at (U.M.); alexander.stadlmann@boku.ac.at (A.S.)

**Keywords:** energy absorption, impact loading, solid beech wood, solid birch wood, temperature-effects

## Abstract

In order to use wood for structural and load-bearing purposes in mechanical engineering, basic information on the impact behaviour of the material over a wide temperature range is needed. Diffuse porous hardwoods such as solid birch wood (*Betula pendula*) and solid beech wood (*Fagus sylvatica*) are particularly suited for the production of engineered wood products (EWPs) such as laminated veneer lumber (LVL) or plywood due to their processability in a veneer peeling process. In the frame of this study, solid birch wood and solid beech wood samples (300 × 20 × 20 mm^3^) were characterised by means of an impact pendulum test setup (working capacity of 150 J) at five temperature levels, ranging from −30 °C to +90 °C. The pendulum hammer (mass = 15 kg) was equipped with an acceleration sensor in order to obtain the acceleration pulse and deceleration force besides the impact bending energy. In both solid birch wood and solid beech wood, the deceleration forces were highest at temperatures at and below zero. While the average impact bending energy for solid birch wood remained almost constant over the whole considered temperature range, it was far less stable and prone to higher scattering for solid beech wood.

## 1. Introduction

The use of wood in the field of mechanical engineering, such as the automotive or aviation industry, has a long history. Wood as an engineering material is widely available, shows low density and low costs, but also high specific strength and elastic properties (see Baumann et al. [1], Müller et al. [2], Aguilera and Davim [3] and Fridley [4]). According to Küch [5], Konnerth [6] and Müller et al. [7], wood species such as pine, ash, spruce and fir are especially favoured for aircraft components from solid wood in the early decades of aviation history. However, unprotected solid wood components that are exposed to weathering show some drawbacks, such as a lack of dimensional stability and also a certain susceptibility to degradation, especially if water is trapped inside the construction (see Küch [5] and Falconer and Rivas [8]). One possible protective measure is the impregnation of solid wood with synthetic resins, e.g., phenolic resins. However, this often results in an uneven distribution of the impregnation substance, especially when it comes to larger cross-sections. In order to overcome this issue, as well as the problem of a relatively high scattering of the mechanical properties in solid wood, veneer-based products such as laminated veneer lumber (LVL) and plywood or cross-banded LVL were introduced (see Küch [5], Kraemer [9] and Forest Products Laboratory [10]). According to Cakiroglu [11], diffuse-porous hardwoods such as birch or beech are especially suited for the production of such peeled veneers due to their relatively uniform density distribution within and across the annual rings. Because of their processability, favourable mechanical properties and availability, birch wood and beech wood are of particular interest for applications in mechanical engineering.

Besides strength and elastic properties, dimensional stability and environmental resistance, another important property for materials exposed to structural and impact loads are its toughness or energy absorbing capability. One relatively old but widespread testing method is the impact pendulum test, which was developed by Charpy at the beginning of the 20th century (see Bayraktar et al. [12]). The test determines the amount of absorbed energy by a notched specimen under impact bending loads during fracture, indicative of its toughness. This energy absorbing capability of a material is usually specified in relation to the cross-sectional area of the sample and is called impact bending energy or impact bending strength. In this study, the terminus impact bending energy is used. Opposed to the notched specimens generally used for testing isotropic materials, e.g., metals or plastics, wood samples such as the geometry of the specimen in ISO 13061 [13] are often unnotched.

In materials that can be assumed as isotropic, the crack resistance to crack growth is almost insensitive to material orientation. The initial and the propagated fracture plane are, therefore, mostly identical. Wood, however, can be considered highly orthotropic. The crack will grow along planes with the least crack growth resistance. In the impact pendulum test, the specimen axis of the wood sample is oriented parallel to the grain or in the longitudinal (L) direction. Therefore, the potential orientation of the initial crack plane is the longitudinal-radial (LR) or longitudinal-tangential (LT) plane. However, there is little chance that the crack will stably grow in the radial (R) or tangential (T) direction, i.e., across the fibre, as fracture resistance is much lower along the fibre, i.e., in the L direction. Therefore, pre-notching is omitted in LR and LT samples. This means that the outcomes encompass not only the energy for fracture propagation but also for initiation. It also means that the outcomes are almost always not only the results of one single fracture system (plane).

Through add-ons, such as, e.g., piezoelectric force transducers in the specimen supports (e.g., Kollmann [14]), additional information, such as reaction force characteristics over the impact time, were obtained. Kollmann concluded that it took about 0.8 ms to 1.4 ms for the more brittle samples (solid pine wood, solid ash wood with low toughness and beech LVL) to reach their maximum force level. On the other hand, samples of more ductile material (solid ash wood with high toughness and solid hickory wood) needed about 2.0 ms to 2.5 ms.

Alternatively, the force characteristics can be determined by strain gauges in the supports as well as in the pendulum hammer (see Bröker and Salamon [15]), allowing to distinguish incoming and reaction forces. The authors report a delay of about 0.3 ms between the initial rise in reaction and the incoming force, which can be explained by the inertia of the sample but also by its finitely high sound velocity. The force–time signals of both the hammer and the supports are characterised by an initial peak followed by an increasing and oscillating force signal. After reaching the maximum force, a sudden softening is observed almost simultaneously at the hammer and the supports. Bucar and Merhar [16] equipped their pendulum hammer with an acceleration sensor. They determined the pendulum deceleration and the impact bending strength. A similar approach was used by Baumann et al. [17], who tested component size solid birch wood samples over a wide temperature range (−30 °C to +90 °C) under quasi-static and dynamic loading. In the case of the dynamic test configuration, acceleration sensors were mounted on the impactor as well as on the two supports of the sample. According to their study, the maximum force level is significantly decreasing with increasing temperature, while the energy-absorbing capability was almost constant over the whole temperature range for the dynamic tests. Kollmann [14] pointed out that the impact bending energy of solid pine wood with moderate moisture content (*u*) of 12% is highest at a temperature of −60 °C and significantly decreases with the increasing temperature down to the lowest point, which is roughly between −30 °C and −10 °C. With a further temperature increase up to +20 °C, the impact bending energy is marginally increasing again.

Regarding the sensitivity of the impact bending energy to the moisture content, Ghelmeziu [18] and Niemz [19] showed that the values for solid beech wood and some coniferous woods are highest at a moisture content between 0% and 5%. Then the impact bending energy values decrease with increasing moisture content before levelling off at roughly 12% to 25%. On the other hand, Krech [20] pointed out that only the force level of solid beech wood decreases with increasing moisture content while the deformability increases, indicating that the impact bending energy is insensitive to moisture content. According to Kollmann [14], the impact bending energy of solid yellow birch wood also remains almost constant in a moisture range between 0% and 10%.

The goal of the present work is to study and compare the influence of temperature (−30 °C to +90 °C) on the impact bending energy as well as the related force–deformation characteristics of solid birch wood and solid beech wood.

## 2. Materials and Methods

### 2.1. Wood Species, Quality and Test Matrix

Below, the terms “birch”, “birch wood” and “solid birch wood” are used synonymously. The same applies to other wood species, i.e., beech. For the sample generation visually graded, air-dried birch (*Betula pendula*) and beech (*Fagus sylvatica*) planks derived from J. u. A. Frischeis GmbH, Stockerau, Austria were used. In order to ensure a clear wood grading, which means that specific growth features and drying cracks are avoided, sampling rules according to ISO 3129 [21] were applied. The geometry of the samples for the impact pendulum tests was chosen following the ISO 13061 [13] with a dimension of 300 × 20 × 20 mm^3^ (length × width × thickness). Thereby, the length of the sample was oriented parallel to the fibre direction. For the width and thickness dimensions, no clear distinction between radial and tangential direction was considered (compare Sell [22] and Wagenführ [23]). This is due to the fact that both beech and birch wood shows no significant difference between the LR and the LT direction when it comes to the mean toughness or mean impact bending energy (see Boruvka [24]). The considered temperature series ranged from −30 °C to +90 °C with a nominal increment of 30 °C [25]. Each of these test configurations was repeated with a total of *n* = 10 samples.

### 2.2. Sample Conditioning

The samples were preconditioned at a reference climate (+20 °C and 65% relative humidity) according to ISO 554 [26] in order to reach an equilibrium moisture content of roughly 12%. Afterward, they were conditioned according to their individual temperature level with a laboratory freezer, a climate chamber or a kiln cabinet. In the case of the temperature series at –30 °C, the samples were preconditioned in the laboratory freezer at −50 °C for 3 h. This overcooling was necessary to compensate for the temperature change between the removal from the freezer and the actual test area. In order to minimise unwanted cooling or heating, the samples were transported in a self-made portable thermo-box. The core temperature was measured immediately prior to the tests with a thermometer PCE-T390 combined with a temperature sensor of type K (both from PCE Deutschland GmbH, Meschede, Germany). A small hole was predrilled in the sample to accommodate the temperature sensor. The temperature series, which was tested at 0 °C, required preconditioning in the climate chamber at −14 °C for two hours. In the case of the series at +30 °C to +90 °C, samples were preconditioned in a kiln cabinet. The preconditioning of the +30 °C series took one hour at +34 °C, the +60 °C series two hours at +68 °C and the +90 °C test series needed three hours at +96 °C.

The density was calculated according to ISO 13061 [27] and Equation (1) on samples with a dimension of 20 × 20 × 20 mm^3^. The density samples were extracted immediately after the tests and in the vicinity of the failure zone.

(1) $$\rho_{u}=\frac{m_{u}}{V_{u}\;}\;\left(\frac{\mathrm{kg}}{m^{3}}\right)$$

where *ρ_u_* is the density at a certain moisture content *u*, *m_u_* is the mass of wood and *V_u_* is the volume of the wood.

The moisture content was evaluated according to ISO 554 [26] and Equation (2). It is determined by the mass of the sample after the impact test (*m_u_*) and the mass after the drying process to complete the oven-dry state in a kiln (*m_od_*).

(2) $$u=\frac{m_{u}-m_{od}}{m_{od}\;}\times 100\;\left(\%\right)$$

where *u* is the moisture content, and *m_od_* is the mass of the oven-dry wood.

In order to properly compare the temperature effect on the mechanical behaviour of the impact bending samples, matched sample sets were established. This was performed with respect to the density on the one hand but also as far as possible for the moisture content.

### 2.3. Testing Device and Measurement Technology

**Figure 1:**
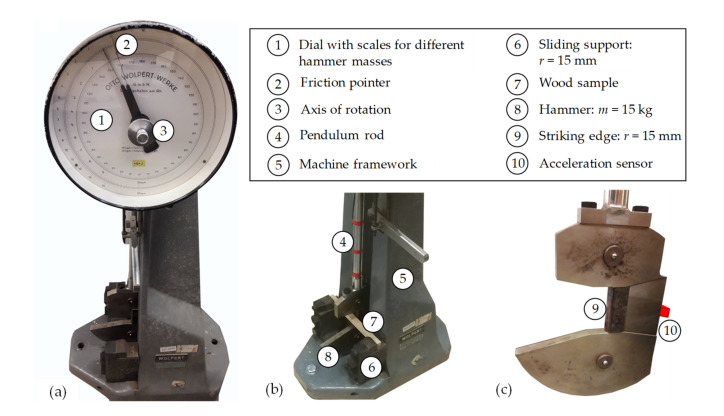
Overview of the impact pendulum testing device from the front (**a**), oblique view of the bottom part including a wood sample (**b**) and detailed view of the hammer head including an acceleration sensor on the back side (**c**).

The tests were conducted in accordance with ISO 13061 [13] using a standardised impact pendulum testing device (Wolpert GmbH, Vienna, Austria) shown in Figure 1. The output of such devices in their standard configuration is either the energy absorption of a sample or its impact bending energy (see Stadlmann et al. [28]). In order to derive information on the occurring force levels, the device was equipped with a uniaxial acceleration sensor, “Model 52”, with a capacity of up to 2000 g (TE connectivity, Hampton, NY, USA). The acceleration sensor was applied on the backside of the hammer opposite to the striking edge (see Figure 1c). It was sampled with a rate of 20 kHz using the amplifier “Minidau” (Kayser-Threde GmbH, Munich, Germany). In addition to the information from the acceleration sensor, high-speed videos with a sampling rate of 1200 frames per second were made using the “Exilim Pro EX-F1” (Casio Europe GmbH, Norderstedt, Germany).

**Figure 2:**
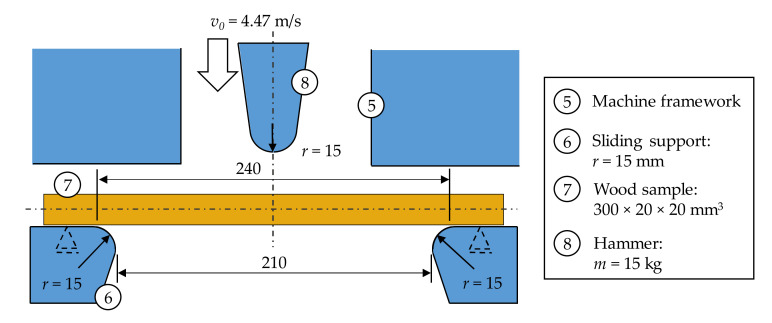
Boundary conditions and geometric dimensions of the wood sample within the impact pendulum testing device.

The impact pendulum testing device can be equipped with different hammer masses. In the present study, a hammer mass of 15 kg, which results in a working capacity of 150 J, was used. This potential energy of *E*_0_ = 150 J leads to an initial hammer velocity of *v*_0_ = 4.47 m/s immediately prior to impact. The striking edge of the hammer, as well as the sliding supports, have a radius of 15 mm, while the centre distance between the two supports is 240 mm with a clear passage width of 210 mm (see Figure 2).

In order to derive a force–time curve from the acceleration signal, additional data processing steps were necessary: the acceleration signal was trimmed from *T*_0_, the time of impact, to *T*_0_ + 10 ms. Furthermore, the signal was filtered with a CFC 1000 low-pass Butterworth filter and converted to a force by assuming an impact mass of *m* = 15 kg.

The calculation of the associated deformation (w) is based on the hammer velocities prior to impact *v_0_* and at the point of complete separation of the sample *v_1_*, whereby the velocity *v_1_* is calculated according to Equation (3).

(3) $$v_{1}=\sqrt{\frac{\left(E_{0}-\Delta E\right)\times 2}{m}}\;\left(\frac{m}{s}\right)$$

where *v*_1_ is the hammer velocity at sample failure, *E*_0_ is the kinetic energy of the hammer prior to impact, and Δ*E* is the measured energy according to the impact pendulum testing device

Between the velocities *v*_0_ and *v*_1_, a linear gradient was assumed. The determined velocity profile was multiplied with the time signal in order to determine the related deformation.

Therefore, a reasonable assumption resulted in a maximum deformation error of 3% in the first 20 mm in comparison to the double-integration of the acceleration signal. The method with the energy-based velocities was preferred over the double-integration method because it is less error-prone to acceleration overshoots caused by unwanted vibrations. Apart from the determination of the impact bending energy with the standard method, it was also derived by integrating the force–deformation curve, which is called the deceleration method. The described evaluation steps were performed using the software “Diadem 2018” (National Instruments, Austin, TX, USA).

## 3. Results and Discussions

### 3.1. Temperature, Moisture and Density

Temperature, moisture content and density are major influencing factors on the impact of bending energy as well as on the material strength (see Kollmann [14], Bucar and Merhar [16], Baumann et al. [17], Niemz [19] and Burmester [29]). An overview of the obtained moisture content and density values at the tested temperature levels is given in Table 1.

It can be seen that the moisture level for both birch and beech at −30 °C and 0 °C is almost identical and close to a general reference moisture content of 12%. However, with increasing temperature levels, the related moisture content is drastically decreasing and close to an oven-dry state for the +90 °C samples. This is due to the fact that the kiln does not allow control over humidity, and wood is a material open for diffusion. The diffusion coefficient is quite heat sensitive and even grows exponentially with increasing temperature (see Kollmann [14]). Therefore, it was not possible to study the pure temperature effect without changes in moisture content. Although the preconditioned samples were transported in a portable thermo-box, and the temperature was measured immediately prior to the tests, there was always a certain temperature gradient between the inner core and the outer surfaces. According to Wimmer [30], the situation for the 0 °C samples is especially complex because water that is bonded in the cell walls propagates to some extent into the lumina, where it starts freezing between −6 °C and −2 °C. It is quite likely that the outermost area had already thawed while the inner core was still frozen.

In order to properly compare the densities between the temperature levels, it was necessary to relate them to a reference moisture level of *u*_ref_ = 12%. These reference densities were calculated according to Kollmann [14] and ISO 554 [26]. Thereby, the birch density had to be adjusted by roughly 5.5 kg/m^3^ and the beech density by 7.2 kg/m^3^ for each percent change in moisture content. The mean density values of birch wood reached from 604 kg/m^3^ to 626 kg/m^3^ with coefficients of variation (CVs) between 3.2% and 4.9%. According to the literature, e.g., Wagenführ [23] (*ρ*_12_ = 510 kg/m^3^ to 830 kg/m^3^), the obtained density values are quite common for birch wood. In the case of beech wood, the obtained mean densities ranged from 794 kg/m^3^ to 820 kg/m^3^ and CVs from 2.5% to 4.2%. The density values of beech wood, according to Wagenführ [23], range from 540 to 910 kg/m^3^.

### 3.2. Impact Bending Energy

One major aspect of this study was the determination of the temperature effect on the energy-absorbing capability or the impact bending energy. The valid results of both the standard method and the deceleration method show a quite good correlation with a coefficient of determination of r^2^ = 0.95. However, the deceleration method leads to impact bending energy values which on a mean value level are between 5% and 12% higher than the values obtained from the standard method. These deviations can be partly explained by the simplified assumption of a linear velocity gradient but also due to acceleration overshoots caused by unpreventable vibration.

**Figure 3:**
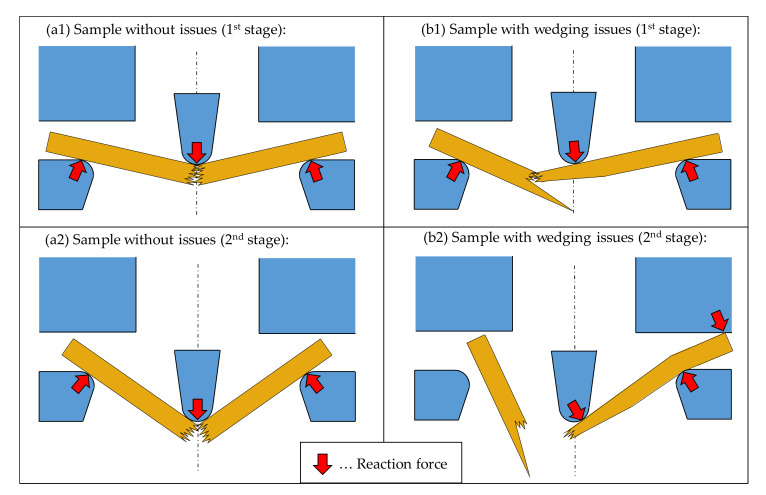
Comparison of the fracture behaviour between a sample without issues (**a1**,**a2**) and a sample with wedging issues (**b1**,**b2**).

A major advantage of the deceleration method over the standard method is its ability to distinguish between the energy amount needed to fracture and separate a sample and possible interference energies, which can occur after the actual test. Such interference energies can be caused by a temporary wedging of sample segments leading to an additional deceleration of the hammer and therefore to invalid results. This phenomenon likely occurs when the sample breaks off-centre. Then the longer half of the specimen may become trapped between the supports and the machine framework. As a result, the hammer decelerates even though the specimen has readily failed. Due to the fact that the whole incident only lasts a few milliseconds, this dissipative mechanism can easily be unnoticed without the aid of the high-speed camera or the acceleration sensor. A comparison of the fracture behaviour between a sample without issues ((a1) and (a2)) and a sample with wedging issues ((b1) and (b2)) is shown in Figure 3.

With the help of the deceleration method and the supplementary high-speed images, it was possible to detect such wedging phenomena in 7% of all tested samples; in detail, three birch wood and four beech wood samples were affected by this phenomenon. On the contrary, the standard method only returns one total value of absorbed energy. This means that the impact bending energy of samples with such wedging issues is either overestimated by this method or, if the error is large enough, they are conspicuous and might be declared as outliers. The inadequacy of the standard method to detect and handle such wedging problems underlines the benefit of coupling this method with the deceleration method.

**Figure 4:**
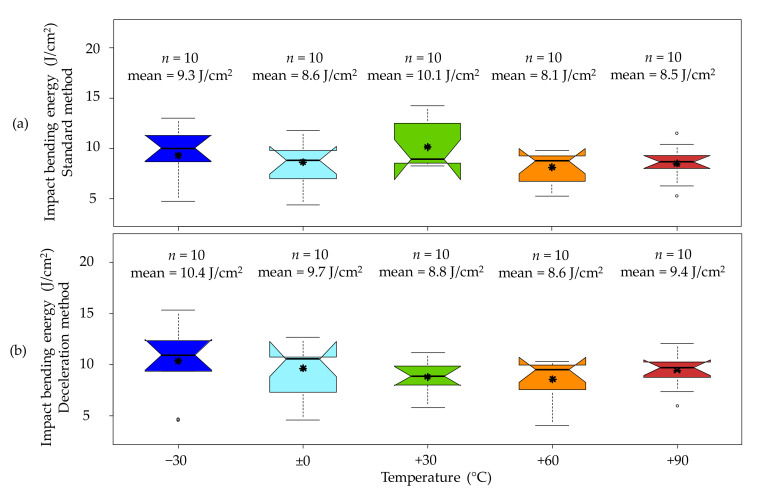
Boxplots of the impact bending energy of birch by temperature level, with the standard method (**a**) and with the deceleration method (**b**). * An interpretation aid concerning the boxplots can be found in Appendix A, Figure A1.

All temperature levels except the +30 °C samples show lower mean values for the standard method than for the deceleration method. The +30 °C mean value is significantly higher for the standard method because all three samples with wedging issues are concentrated in this temperature level. Therefore, interference energies lead to an overestimation of the impact bending energy of the +30 °C samples when using the standard method. If the three samples with wedging issues were excluded from the evaluation, the mean value of the standard method would result in 8.8 J/cm^2^, which (for this specific case with a reduced sample number) is identical to the mean value from the deceleration method. Overall, there is quite a huge scattering of the impact bending energies observed with CVs ranging from roughly 19% to 33% for both methods. However, the median impact bending energy of birch wood remains almost constant over all the considered temperature levels. This trend is quite similar to the results reported by Baumann et al. [17] for birch at temperatures between −30 °C and +90 °C, and Kollmann [14] for pine at temperatures between −30 °C and 20 °C. Figure 4 shows boxplots of the impact bending energy for birch wood by temperature levels, first with the standard method and second with the deceleration method.

**Figure 5:**
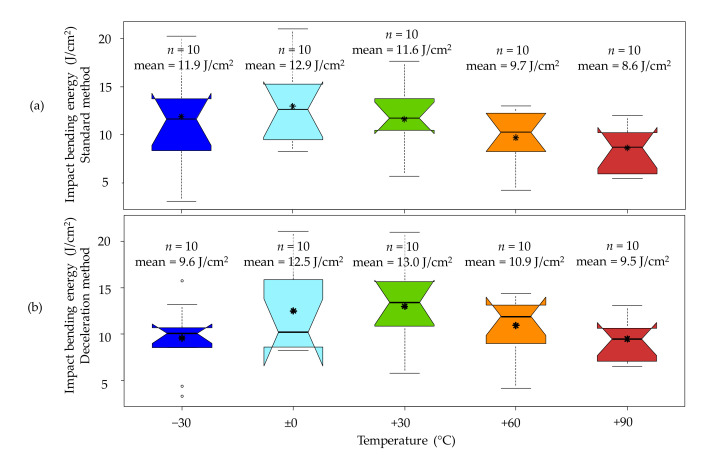
Boxplots of the impact bending energy of beech by temperature level, with the standard method (**a**) and with the deceleration method (**b**). * An interpretation aid concerning the boxplots can be found in Appendix A, Figure A1.

In the case of the impact bending energy values from solid beech wood, which are shown in Figure 5, the coefficient of determination (r^2^) between the standard method and the deceleration method is 0.96 for the valid results. Similar to the birch wood samples, the mean impact bending energy of the beech wood samples is between 6% and 12% higher for the deceleration method than for the standard method. Only the temperature levels −30 °C and 0 °C, which include three and one samples, respectively, with wedging issues, show higher values in the standard method.

The scatter in the impact bending strength values of beech is higher than before on the birch wood with CVs between 25.4% and 44.6%. Contrary to birch, the median impact bending energy values of beech was not as constant over the tested temperature levels. At elevated temperatures (+60 °C and +90 °C), clearly, a decreasing trend with increasing temperature can be observed. This behaviour is comparable to the findings from Reiterer [31], who studied the mode I fracture behaviour of beech in a temperature range between +20 °C and +80 °C. According to his study, the specific fracture energy *G*_f_, which is the integral of the force–displacement curve divided by the fracture area, shows an almost linear decrease with increasing temperature. Moreover, at −30 °C, the mean impact bending energy is rather low, which can be explained by a relatively speedily softening.

A comprehensive list of the essential statistics of birch and beech according to the standard method and the deceleration method, as well as a legend to the boxplots can be found in Appendix A. Table 2 provides an overview of mean impact bending energy values from the literature at a reference climate (+20 °C and 65% relative humidity) according to ISO 554 [26]. The mean values from the literature show a rather large scatter ranging from 7.5 J/cm^2^ up to 17.5 J/cm^2^ for birch wood and from 8.0 J/cm^2^ up to 12.0 J/cm^2^ for beech wood. When calculating an average mean impact bending energy from these sources, it results in 9.6 J/cm^2^ for birch while it is 7.8 J/cm^2^ for beech. Among the conducted experiments, the temperature level of +30 °C comes closest to the literature values at reference climate and is, therefore, best suited for comparison. The mean values of the birch samples at +30 °C are 23.6% lower for the standard method with the excluded wedging samples and the deceleration method than the average value from the literature (8.8 J/cm^2^ vs. 11.5 J/cm^2^). In the case of the beech wood samples at +30 °C, the impact bending energy is 11.5% higher for the standard method and 25% higher for the deceleration method than the average mean value from the literature (11.6 J/cm^2^ and 13.0 J/cm^2^ vs. 10.4 j/cm^2^).

### 3.3. Force–Deformation Behaviour and Fracture Energy

**Figure 6:**
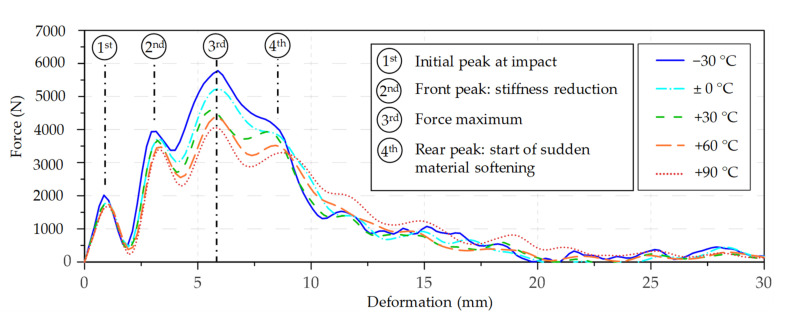
Force–deformation diagram of the mean value curves of birch wood by temperature level.

Figure 6 shows mean value curves of the force–deformation relationship of birch wood for each temperature level. The mean value curves were calculated from the mean gradient of the single test curves. When analysing the force signal, it can be roughly divided into four main stages: the first stage can be described as the initial peak at the impact of the hammer. According to Bröker and Salamon [15], who also equipped their impact pendulum testing device with additional sensors, the initial peak is due to an acceleration shock. The acceleration shock causes the hammer to temporarily lose contact with the sample, leading to a sudden force drop. After the hammer makes contact with the sample for the second time, the force level increases again. The second peak is roughly twice as high as the first one and marks the end of a relatively high initial stiffness. After the second peak, there is a gentle setback followed by a third, final increase, however at a lower gradient, up to the maximum force. This lower gradient between the second and third peak can be explained by successive softening under longitudinal compression. The mean sample deformation when reaching the maximum force level is roughly 6 mm, which is quite comparable to the deformation values of 7 mm up to 9 mm determined by Bröker and Salamon [15]. The fourth peak is significantly lower than the maximum peak and about as high as the second peak. Depending on the temperature level, the fourth peak is more or less pronounced. At lower temperatures (−30 °C and 0 °C), it is harder to discern than at elevated temperatures (+30 °C to +90 °C). Past the fourth peak, a sudden drop of the force signal, likely related to a relatively fast material softening, can be observed.

**Figure 7:**
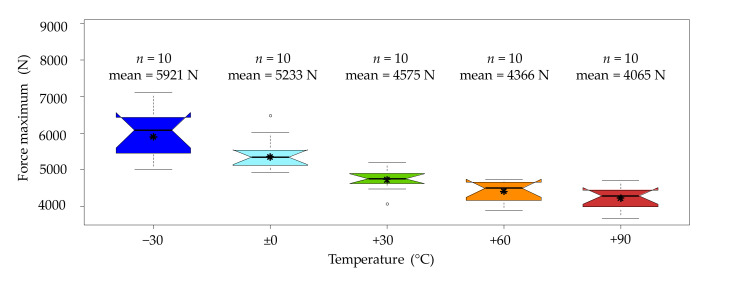
Boxplots of the force maximums from the birch wood tests by temperature level. * An interpretation aid concerning the boxplots can be found in Appendix A, Figure A1.

According to the mean value curves shown in Figure 6, there is almost no temperature effect until the second peak; this only becomes apparent in the course of progressive damage after the second peak. It is assumed that this is primarily controlled by the compressibility of the bending compression zone, which is rather hard and stiff at temperatures below zero and becomes softer with increasing temperatures. In order to obtain an idea of the scattering of the mean value curves and to better visualise the temperature effect, a boxplot of the maximum force levels was generated (see Figure 7). It can be clearly seen that the maximum force level is highest at −30 °C and significantly decreases with increasing temperature. The CV ranges from 6.3% at +60 °C up to 10.3% at −30 °C. This is significantly lower than the CV of the impact bending energy (19% to 33%).

**Figure 8:**
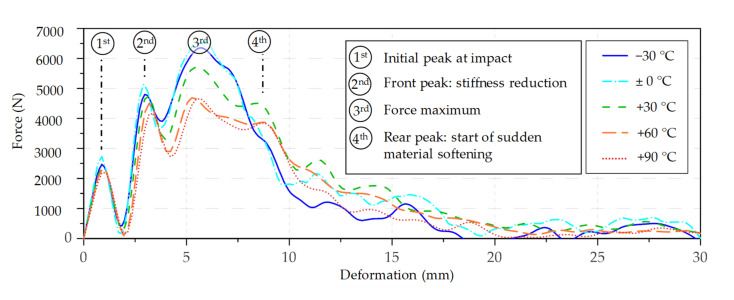
Force–deformation diagram of the mean value curves of beech wood by temperature level.

The force–deformation characteristics of the beech wood samples are quite comparable to the behaviour of the birch wood before, although the mean force level is slightly higher (see Figure 8). The maximum force of all mean curves is also reached at roughly 6 mm deformation. For both the beech and the birch wood samples, it took about 1.4 ms to 1.9 ms to reach the maximum force level, which is quite similar to the values reported by Kollmann [14] (see Chapter 1). Further, it can be stated that the difference between the second peak and the third peak is far less pronounced for the higher temperature levels (+60 °C and +90 °C) than for the lower temperature levels (−30 °C and 0 °C). This can be interpreted as an indicator for a more brittle failure behaviour of beech wood compared to birch wood.

**Figure 9:**
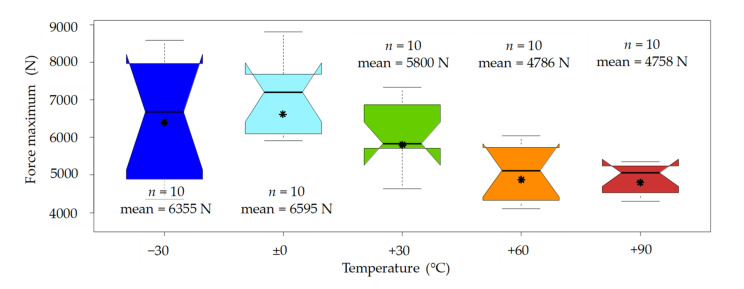
Boxplots of the obtained force maximums from the beech wood tests by temperature level. * An interpretation aid concerning the boxplots can be found in Appendix A, Figure A1.

As with the birch wood samples, beech wood also shows a significant temperature effect when it comes to the force level. The highest maximum force level was observed at −30 °C and 0 °C, with a slightly higher median and mean value at 0 °C. The lowest values were observed at +60 °C and +90 °C. It should be noted that the median values between these two temperature levels were almost identical (5121 N vs. 5062 N). However, the CV of the maximum force levels was significantly higher than with the birch wood samples, ranging from 8.5% at +90 °C to 23.7% at −30 °C (see Figure 9) A comprehensive list of the essential statistics regarding the force maximum (third peak) of birch and beech can be found in Appendix B.

The higher force level at the lower temperature levels can be explained by the supporting effect of the frozen water (see Wimmer [30]). Due to the fact that both the −30 °C as well as the 0 °C samples were overcooled during the preconditioning phase, it can be assumed that in both sample sets, at least the inner core was still frozen during the test. However, Zhao et al. [34] pointed out that the frozen water restricts the deformability of the molecular structure and, therefore, leads to a reduction in ductility. This can also be seen by the slightly steeper softening curve of the present samples. According to Gerhards [35], an increase in temperature leads to a decrease in the material strength, whereby tensile stress is less affected than the compressive strength. This is due to the fact that cellulose, which is primarily responsible for the tensile strength, is not as sensitive to temperature as lignin and hemicellulose, which are primarily responsible for the compression strength.

In both birch and beech, the CV of the maximum force was much smaller than that of the impact bending energy (*E*_tot_). This gives an indication that the crack initiation energy (*E*_init_), which is the integrated energy before the maximum force, should have a lower CV than the crack propagation energy (*E*_prop_), which is the integrated energy after the maximum force (compare Smith et al. [36]). An evaluation of these values showed that the CV of the mean *E*_init_ values ranged from 6.3% to 24.2%, while the CV of the mean *E*_prop_ values was between 28.4% and 59.9% (see Table 3). It can be further seen that the relation between *E*_prop_ and *E*_init_ grows with increasing temperature, which is due to the more ductile softening behaviour at elevated temperatures (compare Figure 6 and Figure 8).

## 4. Conclusions

This study shows that the impact bending energy determined through the standard (change in potential energy) method and the deceleration method correlates well (coefficient of determination r^2^ = 0.95 for birch and r^2^ = 0.96 for beech). However, the mean results of the deceleration method were 5% to 12% higher than those of the standard method. The major advantage of the deceleration method over the standard method is that it allows identifying samples where wedging occurred that would otherwise have distorted statistics calculated from such samples. Therefore, the deceleration method can be considered as a good complement to the standard method. It was further shown that the impact bending energy of beech wood is not as constant as that of birch wood over the temperature levels (−30 °C to +90 °C). Further, the coefficient of variation in the impact bending energy was also significantly higher for beech (CVs between 25.4% and 44.6%) than for birch (CVs between 19.0% and 33.0%).

In terms of the force–deformation characteristics, the behaviour of birch and beech wood was quite similar. Both wood species showed a pronounced decrease in the maximum force level with increasing temperature. As with the impact bending energy, the coefficient of variation in the maximum force was significantly higher for beech (CVs between 8.5% and 23.7%) than for birch (CVs between 6.3% and 10.3%). Overall, the mean maximum force levels of the beech wood samples (4758 N to 6595 N) were slightly higher than those of the birch wood samples (4065 N to 5921 N), which can be partially explained by their higher density. The time to maximum force, as well as the related mean deformation, was almost identical for birch and beech and also for all tested temperature levels. The values for the duration were between 1.4 ms and 1.9 ms, and the mean deformation was roughly 6 mm.

## Figures and Tables

**Table 1:** Overview of the main statistics regarding moisture content and density adapted to reference moisture condition of *u*_ref_ = 12% (CV—Coefficient of variation).

Wood Species	Temperature-Level (°C)	*n* (–)	*u*_mean_ (%)	CV(*u*) (%)	*ρ*_12,mean_ (kg/m^3^)	CV(*ρ*_12_) (%)
Birch	−30	10	10.6	3.7	621	4.5
	0	10	10.7	5.2	614	3.7
	+30	10	8.3	2.7	626	3.8
	+60	10	4.7	5.2	604	4.9
	+90	10	1.0	16.4	613	3.2
Beech	−30	10	10.7	3.5	794	2.5
	0	10	10.4	4.2	820	3.6
	+30	10	8.9	5.6	815	4.2
	+60	10	5.7	9.9	811	2.9
	+90	10	1.4	22.7	800	2.3

**Table 2:** Average ranges of impact bending energy values for small clear wood specimens of birch and beech.

Material Value	Authors			
	Sell [22]	Wagenführ [23]	Becker et al. [32]	Kretschmann [33]
Impact bending energy birch (J/cm^2^)	7.5 to 10 *	10 **	-	14.1 to 17.5 ***
Impact bending energy beech (J/cm^2^)	8 to 12 ^+^	10 ^+^	11.2 ^+^	-

* Betula verrucosa; ** Betula pendula Roth; *** Betula alleghaniensis; ^+^ Fagus sylvatica.

**Table 3:** Overview of the crack initiation energy, the crack propagation energy, the total energy, mean values and their CVs based on the deceleration method (CV—Coefficient of variation).

Wood Species	Temperature- Level (°C)	*n* (−)	*E*_init_ (J/cm^2^)	CV(_init_) (%)	*E*_prop_ (J/cm^2^)	CV(_prop_) (%)	*E*_tot_ (J/cm^2^)	CV(_tot_) (%)
Birch	−30	10	4.5	14.1	5.9	52.6	10.4	33.2
	0	10	3.9	8.1	5.8	42.2	9.7	26.0
	+30	10	3.4	8.8	5.4	29.2	8.8	18.8
	+60	10	3.3	8.1	5.3	34.8	8.6	23.3
	+90	10	3.0	6.3	6.4	28.4	9.4	19.3
Beech	−30	10	4.6	24.2	5.0	55.6	9.6	38.3
	0	10	5.0	12.6	7.5	59.9	12.5	37.2
	+30	10	4.5	15.0	8.5	49.1	13.0	35.4
	+60	10	3.8	13.8	7.1	42.6	10.9	31.5
	+90	10	3.5	8.5	6.0	37.5	9.5	25.4

**Figure A1:**
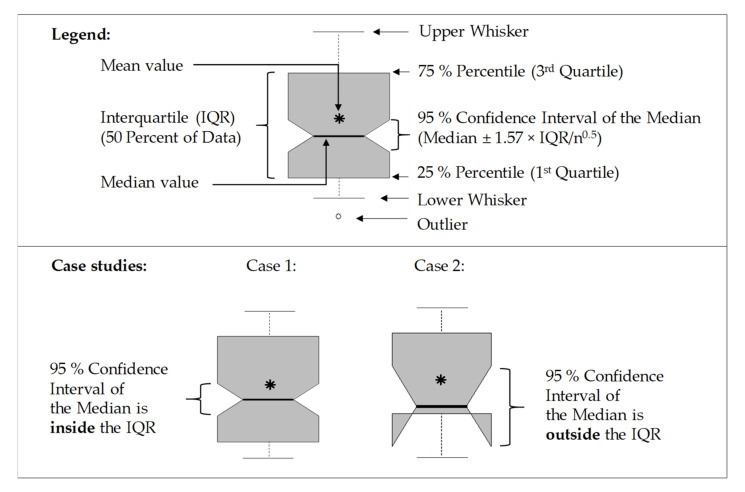
Elements of a Boxplot with notches including case studies.

## Data Availability

All the data is available within the manuscript.

## Abbreviations

EWP	Engineered wood product
LVL	Laminated veneer lumber
L	Longitudinal direction
R	Radial direction
T	Tangential direction
CV	Coefficient of variation
r^2^	Coefficient of determination

## Appendix A

**Table A1:** Essential statistics of birch according to the standard method and the deceleration method: mean impact bending energy (*E*_tot,mean_), median impact bending energy (*E*_tot,median_), coefficient of variation of the impact bending energy (CV(_tot_)), minimum impact bending energy (*E*_tot,min_) and maximum impact bending energy (*E*_tot,max_). Values in parentheses are statistics without invalid results.

Testing Method	Temperature-Level (°C)	*n* (−)	*E*_tot,mean_ (J/cm^2^)	*E*_tot,median_ (J/cm^2^)	CV(_tot_) (%)	*E*_tot,min_ (J/cm^2^)	*E*_tot,max_ (J/cm^2^)
Standard method	−30	10	9.3	10	28.4	4.8	13.0
	0	10	8.6	8.8	24.7	4.4	11.8
	+30	10 (7)	10.1 (8.8)	8.8 (8.8)	22.2 (16.7)	8.3 (8.3)	14.3 (10.1)
	+60	10	8.1	8.8	20.0	5.3	9.8
	+90	10	8.5	8.6	21.3	5.3	11.5
Deceleration method	−30	10	10.4	10.9	33.2	4.6	15.3
	0	10	9.7	10.6	26.0	4.6	12.7
	+30	10	8.8	8.9	18.8	5.8	11.2
	+60	10	8.6	9.5	23.3	4.1	10.3
	+90	10	9.4	9.7	19.3	6.0	12.1

**Table A2:** Essential statistics of beech according to the standard method and the deceleration method: mean impact bending energy (*E*_tot,mean_), median impact bending energy (*E*_tot,median_), coefficient of variation of the impact bending energy (CV(_tot_)), minimum impact bending energy (*E*_tot,min_) and maximum impact bending energy (*E*_tot,max_). Values in parentheses are statistics without invalid results.

Testing Method	Temperature-Level (°C)	*n* (−)	*E*_tot,mean_ (J/cm^2^)	*E*_tot,median_ (J/cm^2^)	CV(_tot_) (%)	*E*_tot,min_ (J/cm^2^)	*E*_tot,max_ (J/cm^2^)
Standard method	−30	10 (7)	11.9 (9.4)	11.6 (9.3)	44.6 (37.0)	3.1 (3.1)	20.3 (13.8)
	0	10 (9)	12.9 (12.0)	12.6 (10.8)	32.7 (28.2)	8.3 (8.3)	21.0 (17.0)
	+30	10	11.6	11.8	30.2	5.8	17.6
	+60	10	9.7	10.3	30.4	4.3	13.0
	+90	10	8.6	8.7	28.3	5.5	12.0
Deceleration method	−30	10	9.6	10.1	38.3	3.3	15.7
	0	10	12.5	10.2	37.2	8.3	21.1
	+30	10	13.0	13.4	35.4	5.8	21.0
	+60	10	10.9	11.9	31.5	4.2	14.4
	+90	10	9.5	9.5	25.4	6.6	13.1

## Appendix B

**Table A3:** Essential statistics regarding the maximum force level (3rd peak) of birch and beech: mean value of the 3rd peak (*F*_3rd,mean_), median value of the 3rd peak (*F*_3rd,median_), coefficient of variation of the 3rd peak (CV(_3rd_)), minimum value of the 3rd peak (*F*_3rd,min_) and maximum value of the 3rd peak (*F*_3rd,max_).

Wood Species	Temperature-Level (°C)	*n* (−)	*F*_3rd,mean_ (N)	*F*_3rd,median_ (N)	CV(_3rd_) (%)	*F*_3rd,min_ (N)	*F*_3rd,max_ (N)
Birch	−30	10	5921	6078	10.3	5002	7103
	0	10	5233	5240	9.0	4908	6489
	+30	10	4575	4596	6.6	4065	5193
	+60	10	4366	4496	6.3	3868	4726
	+90	10	4065	4278	7.6	3673	4696
Beech	−30	10	6355	6674	23.7	4362	8594
	0	10	6595	7194	14.5	5903	8817
	+30	10	5800	5827	13.9	4633	7339
	+60	10	4786	5121	14.7	4093	6047
	+90	10	4758	5062	8.5	4301	5354
